# Measured solar irradiance data for resource assessment at four sites in Geba catchment, Tigray, North Ethiopia

**DOI:** 10.1016/j.dib.2022.107836

**Published:** 2022-01-17

**Authors:** Mulu Bayray, Yacob Gebreyohannes, Hailay Gebrehiwot, Solomon Teklemichael, Anwar Mustefa, Asfafaw Haileslassie, Petros Gebray, Ashenafi Kebedom, Fana Filli

**Affiliations:** Thermal and Energy Systems Chair, School of Mechanical and Industrial Engineering, Ethiopian Institute of Technology-Mekelle, Mekelle University, Ethiopia

**Keywords:** Solar radiation, Pyranometer, Resource assessment, Geba catchment, Ethiopia

## Abstract

Properly recorded solar radiation data are very important in providing accurate information on solar radiation intensity and potential for the application of any solar energy technology. Since such type of data is hardly available in most developing countries like Ethiopia, analysis of temporal and spatial variations of solar radiation is essential for exploring the true potential of a specific area. This scientific data article is, therefore, related to the research work entitled “Temporal and Spatial Solar Resource Variation by Analysis of Measured Irradiance in Geba Catchment, North Ethiopia” (https://doi.org/10.1016/j.seta.2021.101110). In this work, we present the solar radiation measurement data collected for five years (from January 2011 to December 2015) from four sites of the Geba catchment (Dera, May Derhu, Hagere Selam, and Mekelle University) located in the Northern part of Ethiopia. Data were measured at ten-minute intervals using Pyranometers mounted on wind masts. The data was used for the analysis of the temporal variation and spatial distribution performed using MS Excel spreadsheet and Inverse Distance Weight (IDW) method of the ArcGIS software, respectively. Accordingly, the data revealed insight on the solar variation and potential of the catchment and is expected to contribute significantly to further decision-making by governmental and non-governmental agencies, investors, consultants, and project developers. It is also expected to help for future research and solar project implementation directions across similar catchments.

**Specifications Table**

**Table:** 

Subject	Renewable Energy, Sustainability and the Environment
Specific subject area	Solar Resource Assessment
Type of data	Table, Graph, Figure
How data were acquired	Data were acquired from four stations using Davis Vantage Pro 6450 Solar Radiation Sensor that measures total global radiation on a horizontal surface. Data were recorded in the EKO 21 N data logger. The logging set up was at three-second intervals, 200 data points, and recorded the average every ten minutes. Data were downloaded to a Laptop computer for data filter and analysis using an MS Excel Spreadsheet and the Inverse Distance Weight (IDW) method of the ArcGIS software.
Data format	Raw, Filtered, Analysed
Parameters for data collection	Measurement of Global Horizontal Irradiance (GHI)
Description of data collection	Data was collected from regular visits made to the sites. The raw data (Global Solar Irradiance) collected from the data logger was processed in three steps: (i) Data extraction, (ii) Data Qualification and Labeling, and (iii) Data Analysis.
Data source location	Geba Catchment, Tigray, North Ethiopia
Data accessibility	Data is available in a supplementary file and the article
Related research article	Mulu Bayray, Yacob Gebreyohannes, Hailay Gebrehiwot, Solomon Teklemichael, Anwar Mustefa, Asfafaw Haileslassie, Petros Gebray, Ashenafi Kebedom, Fana Filli.“Temporal and Spatial Solar Resource Variation by Analysis of Measured Irradiance in Geba Catchment, North Ethiopia”, Sustainable Energy Technologies and Assessments, 44, 101,110, 2021. (https://doi.org/10.1016/j.seta.2021.101110)

**Value of the Data**

• Data provides insight into the solar potential that exists in the four sites of Geba catchment, Tigray, North Ethiopia.
• The data could be useful to solar energy researchers, solar project developers, governmental and non-governmental agencies, investors, and consultants.
• Data can be compared with other satellite and model-predicted data sets and/or is useful in future monitoring of solar energy projects.
• Data revealed the extent of temporal and spatial variations of solar radiation in the Geba catchment.

## 1 Data Description

Global solar irradiance measurements were taken at ten minutes intervals. The collected data was not complete due to the data gap created as a result of a delayed start or early stop and measurement errors while measurements were taken. These errors were occurring mainly due to maintenance work and the failure of batteries and sometimes the data logger.

From the actual measurement data of the sites in the catchment, the solar radiation data were recorded for five years in Dera, for about three years and seven months in Hagere Selam (HS), for about four years and eight months in May Derhu (MD), and about three years and three months in the Mekelle University (MU) campus site. The measured data in all sites have a total count of 736,324. The start date, end date, and data points for each measurement site are given in Table 1. Following that, correction or removal of errors that may lead to biased and misleading results was carried out. The supplementary material (QLdata_Geba catchment.xls) provides the raw data relative to each repeat used to identify the errors in measurement and calculate the time-series averages. This ten-minute qualified data for 2011 to 2015 was used to calculate the average hourly solar radiation over 24 h as indicated in Fig. 3 to Fig. 7, respectively. The missed data in each site of the catchment were determined and summarized in Tables 2–5. The tables indicate the number of actual recorded data, data gaps, and missed data. The raw data obtained from the MU site had a lot of missing values.

**Table 1:** Summary of raw data measurements.

Site Name	Start Date	End Date	Total Data Points
**Dera**	January 1/2011 at 0:01:38	December 31/2015	261,923
**HS**	January 1/2011 at 0:06:20	July 22/2014 at 17:40:36	168,967
**MD**	January 15/2011	August 9/2015 at 13:04:21	207,043
**MU**	January 1/2011 at 0:02:06	March 23/2014 at 15:21:50	98,391

**Table 2:** Raw data characteristics on annual basis for Dera site.

	Data Gaps					
Year	Delayed Start	Early Stop	Missed Data	Actual Data	Expected Data	Share of Unavailable Data (%)
2011	–	–	0	52,560	52,560	0.00
2012	–	–	0	52,704	52,704	0.00
2013	–	–	1012	51,548	52,560	1.92
2014	–	–	0	52,560	52,560	0.00
2015	–	–	0	52,560	52,560	0.00

**Total**	**0**	**0**	**1012**	**261,932**	**262,944**	**0.04**

**Table 3:** Raw data characteristics on annual basis for HS site.

	Data Gaps					
Year	Delayed Start	Early Stop	Missed Data	Actual Data	Expected Data	Share of unavailable Data (%)
2011	–	–	4564	47,996	52,560	8.68
2012	–	107	–	52,597	52,704	0.20
2013	–	–	10,155	42,405	52,560	19.32
2014	–	23,365	307	28,888	52,560	45.03

**Total**	**0**	**23,472**	**15,026**	**171,886**	**210,384**	**18.29**

**Table 4:** Raw data characteristics on annual basis for the MD site.

	Data Gaps					
Year	Delayed Start	Early Stop	Missed Data	Actual Data	Expected Data	Share of Unavailable Data (%)
2011	2091	–	7	50,462	52,560	4.00
2012	–	–	3	52,701	52,704	0.006
2013	–	26,689	1	25,870	52,560	50.80
2014	5674	–	–	46,886	52,560	10.80
2015	–	20,799	7	31,754	52,560	39.60

**Total**	**7765**	**47,488**	**18**	**207,673**	**262,944**	**21.02**

**Table 5:** Raw data characteristics on annual basis for MU campus site.

	Data Gaps					
Year	Delayed Start	Early Stop	Missed Data	Actual Data	Expected Data	Share of Unavailable Data (%)
2011	–	34,764	–	17,796	52,560	66.14
2012	2685	–	–	50,019	52,704	5.09
2013	–	–	28,049	24,511	52,560	53.36
2014	–	41,898	5002	5660	52,560	89.23

**Total**	**2685**	**76,662**	**33,051**	**97,986**	**210,384**	**53.42**

The compiled diurnal solar radiation data are included as supplemental material (HDA_Geba catchment.xls). Relatively higher radiation values are observed during the months when the sky is characteristically clear (i.e. September to May). The hourly average solar radiation values of a day attained their maximum value almost at noon during these dry months (Fig. 8). Low solar radiation values were observed during the rainy season (i.e. June to August). In most of the diurnal hourly average solar radiation data during the rainy months, a wrinkling curve around noon due to the shadow of the mast has been observed (Fig. 9). Correction of this measurement error was not considered as it was difficult to isolate the influence of the shadow of the mast from that of a decrease in solar radiation due to clouds. Table 6 summarizes the daily solar radiation variation range (minimum and maximum values) for each site and each year. The result from the daily analysis indicates the catchment is endowed with a considerable solar energy resource which varies from around 1.06 kWh/m^2^day in the rainy month of August to 8.04 kWh/m^2^day in the dry month of March.

**Table 6:** The range of daily irradiation values on an annual basis (kWh/m^2^).

Location	Dera		HS		MD		MU Campus	
Year	Min	Max	Min	Max	Min	Max	Min	Max
2011	1.28	8.04	1.06	8.02	1.32	8.04	1.93	8.02
2012	2.13	7.81	1.68	7.40	2.2	7.70	2.30	7.80
2013	2.00	7.80	1.28	7.81	1.80	7.70	2.43	7.85
2014	1.27	7.90	2.73	7.59	2.04	7.70	–	–
2015	1.89	7.63	–	–	1.79	7.73	–	–

The compiled monthly and seasonal data are included in this article. The monthly averaged daily solar radiation is given in Table 7. The table depicts high solar radiation measurements during the dry months and more specifically in April and March. Except for the months in the rainy season (June, July, and August); the average solar radiation is observed to be greater than 5 kWh/m^2^. The lowest average observed was attributed to the high coverage of clouds in these months.

**Table 7:** Monthly average daily solar irradiation (kWh/m^2^).

		Month											
Location	Year	Jan	Feb	Mar	Apr	May	Jun	Jul	Aug	Sep	Oct	Nov	Dec
**Dera**	2011	5.32	6.97	5.96	6.62	5.22	4.64	3.63	4.60	5.66	6.25	5.43	6.12
	2012	6.10	7.01	6.63	6.01	5.80	4.52	3.93	5.00	6.39	6.40	5.43	5.90
	2013	5.49	6.57	6.16	6.24	5.55	4.14	3.58	4.86	5.87	6.07	5.70	5.87
	2014	5.65	6.33	5.36	6.45	5.30	4.92	2.52	4.57	5.66	5.73	5.43	5.73
	2015	6.06	6.55	6.54	6.69	5.09	4.40	4.21	4.44	5.90	6.13	5.38	4.76

**HS**	2011	4.89	6.79	5.57	6.81	5.25	4.23	4.21	3.81	5.19	6.37	4.61	6.11
	2012	6.02	6.78	6.34	5.87	5.65	4.07	3.43	3.91	5.31	6.31	5.44	5.48
	2013	5.37	6.23	6.14	6.35	–	–	3.85	3.73	4.41	–	–	–
	2014	5.42	6.12	6.72	6.54	5.13	4.60	4.62	–	–	–	–	–

**MD**	2011	4.73	7.03	6.13	6.64	5.42	4.00	4.03	3.71	5.39	6.65	5.27	6.14
	2012	6.10	7.08	6.60	5.90	6.02	4.20	3.54	4.31	5.73	6.77	6.15	5.98
	2013	5.64	6.46	6.08	6.21	5.53	4.39	–	–	–	–	–	–
	2014	–	6.01	6.63	6.57	5.46	4.51	4.22	4.52	5.44	6.36	5.68	6.14
	2015	5.86	6.75	6.84	7.29	5.76	4.48	4.57	4.16	–	–	–	–

**MU Campus**	2011	5.39	6.63	6.23	6.90	5.33	–	–	–	–	–	–	–
	2012	6.00	6.80	6.80	6.20	6.80	5.00	3.90	4.10	5.50	6.50	5.90	5.50
	2013	5.57	6.31	6.28	6.56	–	–	–	–	–	5.50	5.91	4.99
	2014	–	–	–	–	–	–	–	–	–	–	–	–

The seasonally averaged daily solar radiations are given in Table 8. The table shows that the seasonal solar radiation values are not noticeably different during winter and spring, while in the summer season the value is lower than the other seasons of the year. The maximum seasonal solar radiation was recorded in the winter and spring seasons. The seasonally averaged daily solar radiation of the catchment varies from around 4.28 kWh/m^2^ in summer to 6.15 kWh/m^2^ in spring.

**Table 8:** Seasonal average daily solar irradiation (kWh/m^2^).

		Season			
Location	Year	Autumn	Winter	Spring	Summer
**Dera**	2011	5.78	6.11	5.93	4.29
	2012	6.08	6.32	6.15	4.48
	2013	5.90	6.00	6.00	4.19
	2014	5.62	5.93	5.69	4.53
	2015	5.81	5.77	6.10	4.35

**HS**	2011	5.48	5.90	5.94	4.06
	2012	5.70	6.10	5.90	3.80
	2013	–	6.26	6.24	3.79
	2014	–	5.65	6.12	4.58

**MD**	2011	5.78	6.15	6.06	3.92
	2012	6.20	6.44	5.17	4.06
	2013	–	6.00	5.90	4.40
	2014	5.85	6.09	6.22	4.42
	2015	–	6.28	6.62	4.48

**MU Campus**	2011	–	5.98	6.56	–
	2012	5.90	6.10	6.60	4.40
	2013	–	5.59	6.51	–
	2014	–	–	–	–

Seasonal solar radiation distribution across the four sites of the catchment is shown in Fig. 10. In autumn (Fig. 10a), the highest interpolated seasonal solar radiation values are observed in the south around the MD site. During this season the values of solar radiation in the catchment are ranging from 5.5 to 5.9 kWh/m^2^ day. Comparatively the lowest estimated value in the catchment is observed around the HS site. In winter (Fig. 10b), the estimated solar radiation distribution map of the catchment indicates that the values vary from a minimum of 5.8 kWh/m^2^ to a maximum value of 6.1 kWh/m^2^. Similar to the autumn season, the highest solar radiation is observed around the MD site and slightly extended North towards the Dera site. In spring (Fig. 10c), comparatively low solar resources were obtained in some parts of North HS and some parts of North-East Dera. However, the relatively higher solar resource is observed in the MU campus site and slightly extended towards MD and Dera sites. The estimated average seasonal solar radiation distribution of spring is ranging from about 5.96 to 6.35 kWh/m^2^. In summer (Fig. 10d), it is observed that the distribution is relatively high around the MU campus site. The estimated average seasonal solar radiation distribution of summer is ranging from 4.04 to 4.36 kWh/m^2^. The average solar radiation value during the summer season is lower since cloud cover is the one affecting the availability of solar radiation intensity. Overall, the result of the study showed the variation of the seasonal average daily solar radiation from around 4.04 kWh/m^2^/day in summer to 6.35 kWh/m^2^/day in spring. The spatial distribution showed a variation of 5 to 7.5% in solar radiation obtained in the catchment stations. Areas in the central part around MU and the southern part around the MD site have relatively higher solar radiation. The total daily average solar radiation for the catchment was found to be 5.6 kWh/m^2^/day, which was equivalent to 2045 kWh/m^2^/year. Considering this calculated annual solar radiation intensity, the catchment's annual technical potential was estimated to be 8.3 PWh.

## 2 Experimental Design, Materials and Methods

### 2.1 Description of sites

**Fig. 1:**
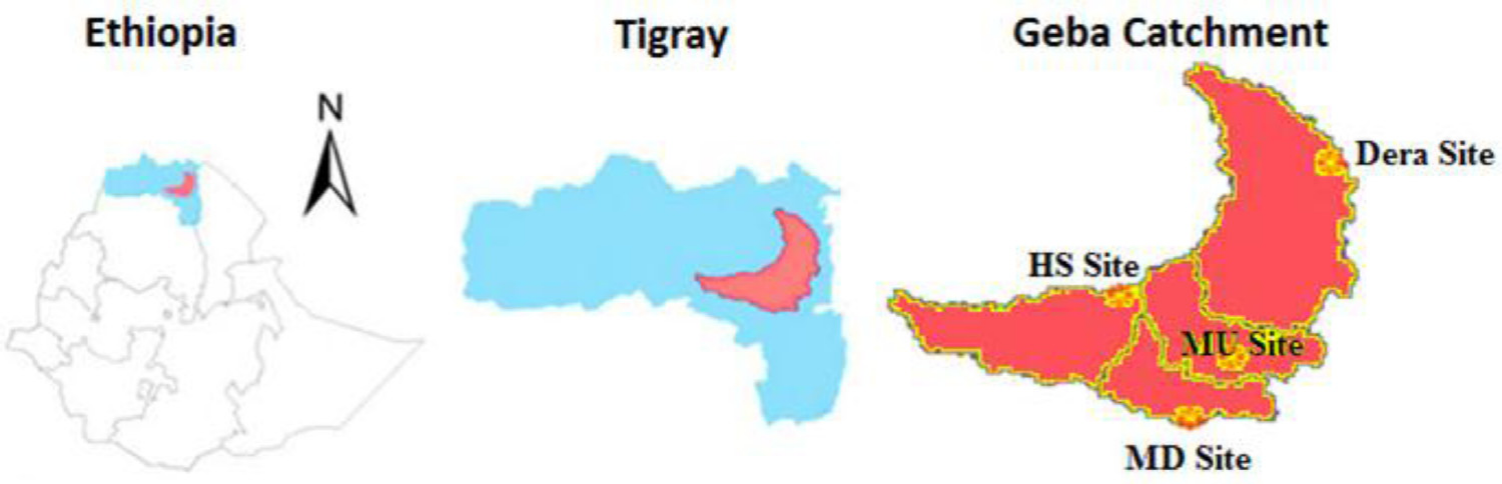
The geographical location of the Geba catchment.

Geba catchment is located in the Tigray region, North Ethiopia located between longitude 38°38′ to 39°48′ East and latitude 13°18′ to 14°15′ North (Fig. 1). The catchment covers an area of nearly 5133 km^2^ with a mean elevation of 2164 m above sea level (elevation varies from 955 to 3295 m) [1,2]. The catchment is extensively cultivated and agricultural activities occur even on many of the steep and stony valley sides [3]. The measurement sites are located at Dera (13.99° N, 39.73° E, 2870 m), HS (13.66° N, 39.19° E, 2628 m), MD (13.29° N, 39.40° E, 2512 m), and MU (13.48° N, 39.49° E, 2208 m).

### 2.2 Experimental setup and instrumentation

**Fig. 2:**
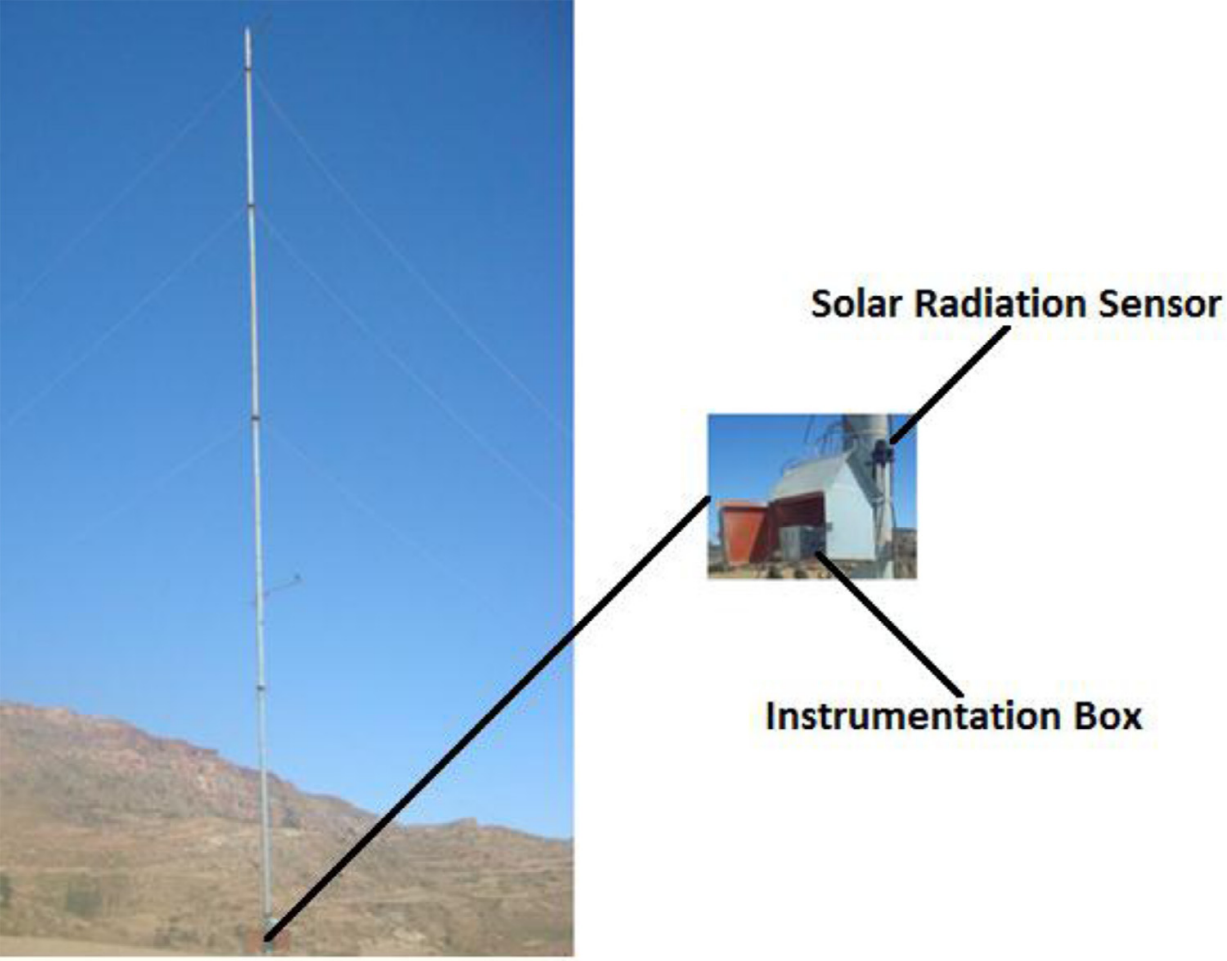
Mast setup and instrumentation (MD site).

The data used in this study was collected by ground measurement devices Pyranometers (Davis model DS6450) installed at wind masts of four stations of the study area. The solar radiometer was installed above the instrument box about 2 m above ground level as shown in Fig. 2. The date of installation and other instrumentation information has been reported in [1].

**Fig. 3:**
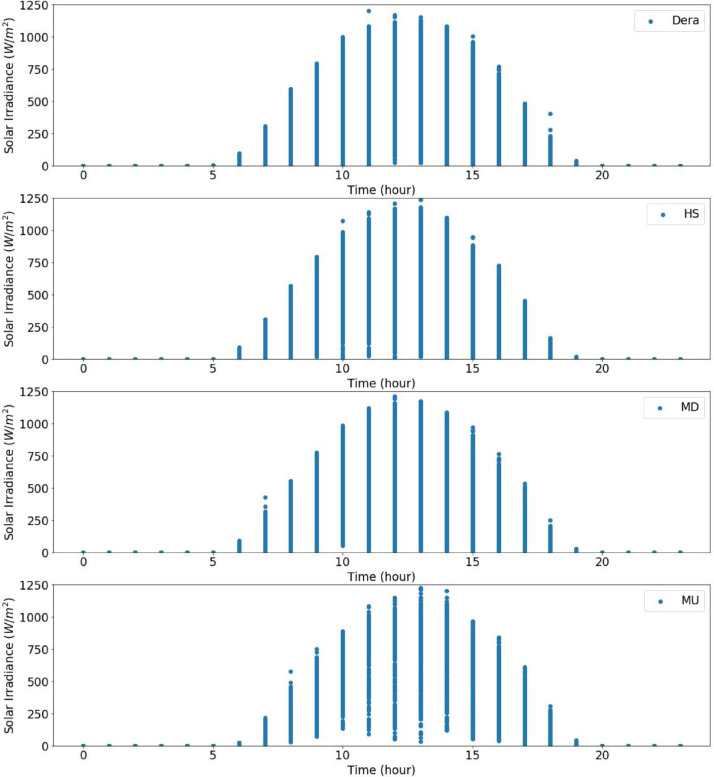
Overall radiation measurement in 2011 over 24 h.

**Fig. 4:**
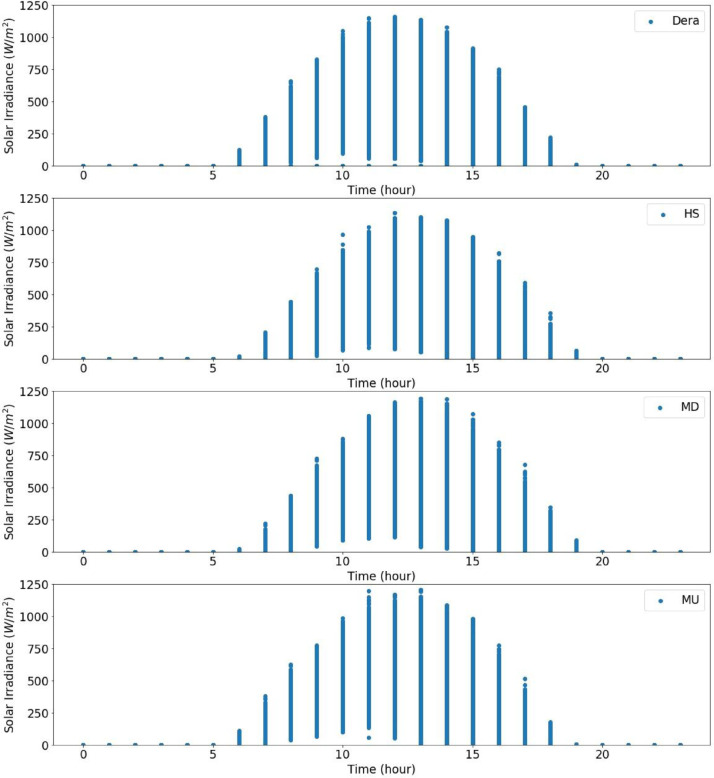
Overall radiation measurement in 2012 over 24 h.

**Fig. 5:**
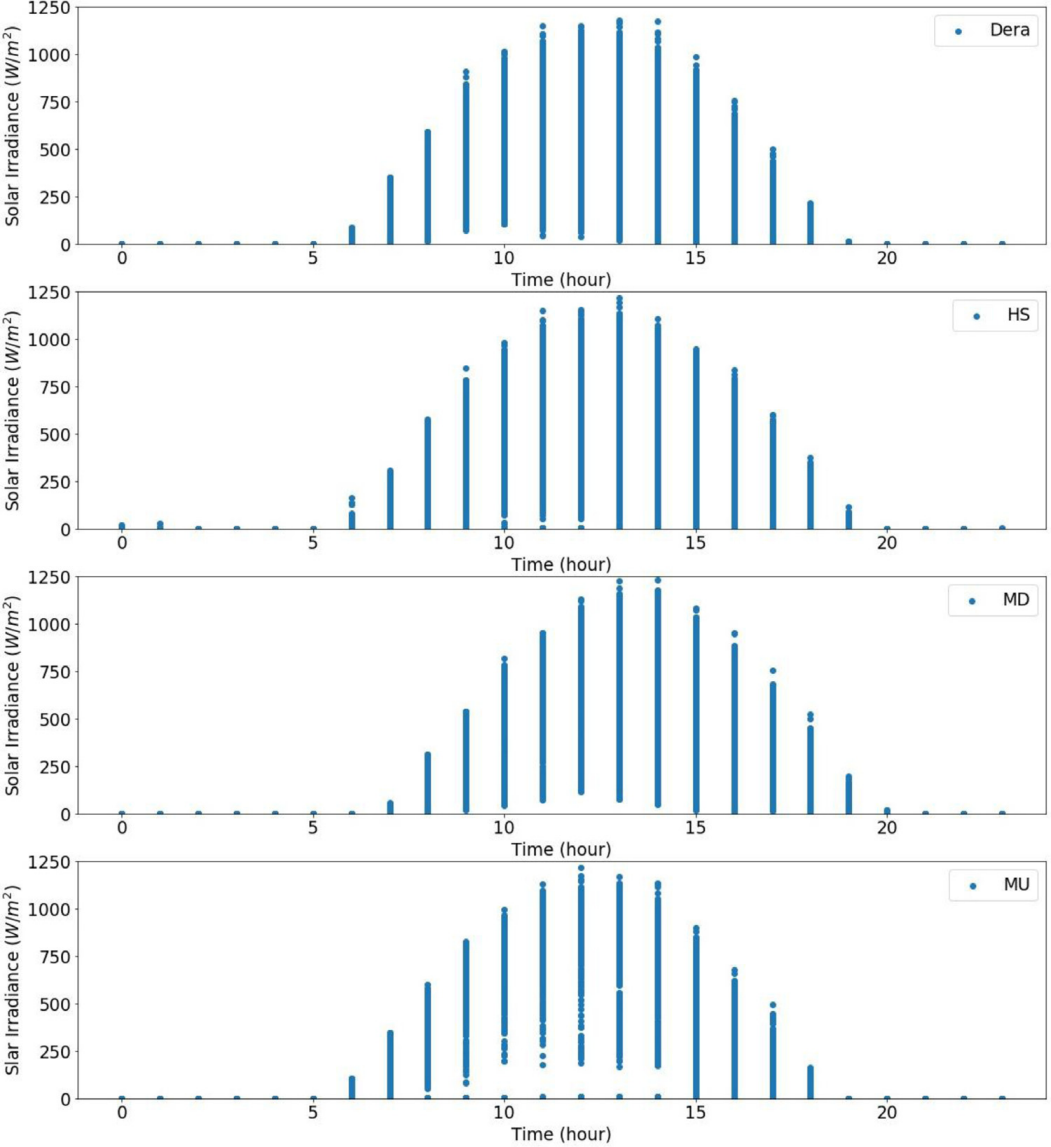
Overall radiation measurement in 2013 over 24 h.

**Fig. 6:**
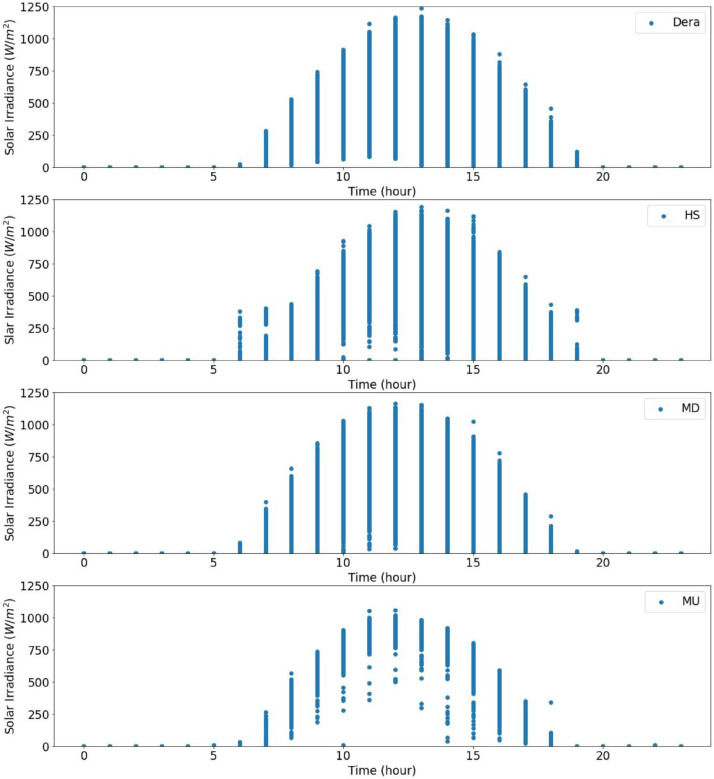
Overall radiation measurement in 2014 over 24 h.

**Fig. 7:**
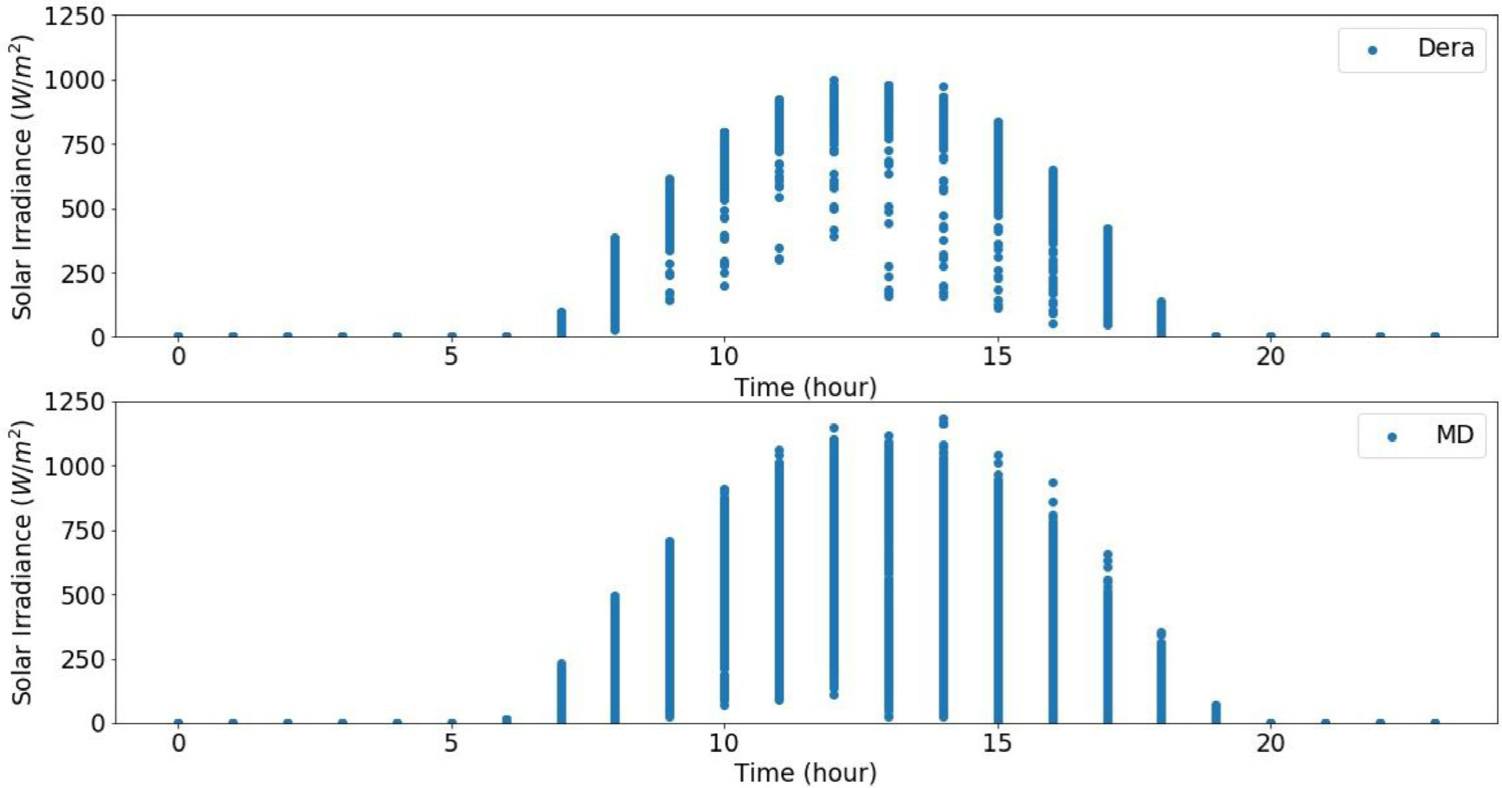
Overall radiation measurement in 2015 over 24 h.

**Fig. 8:**
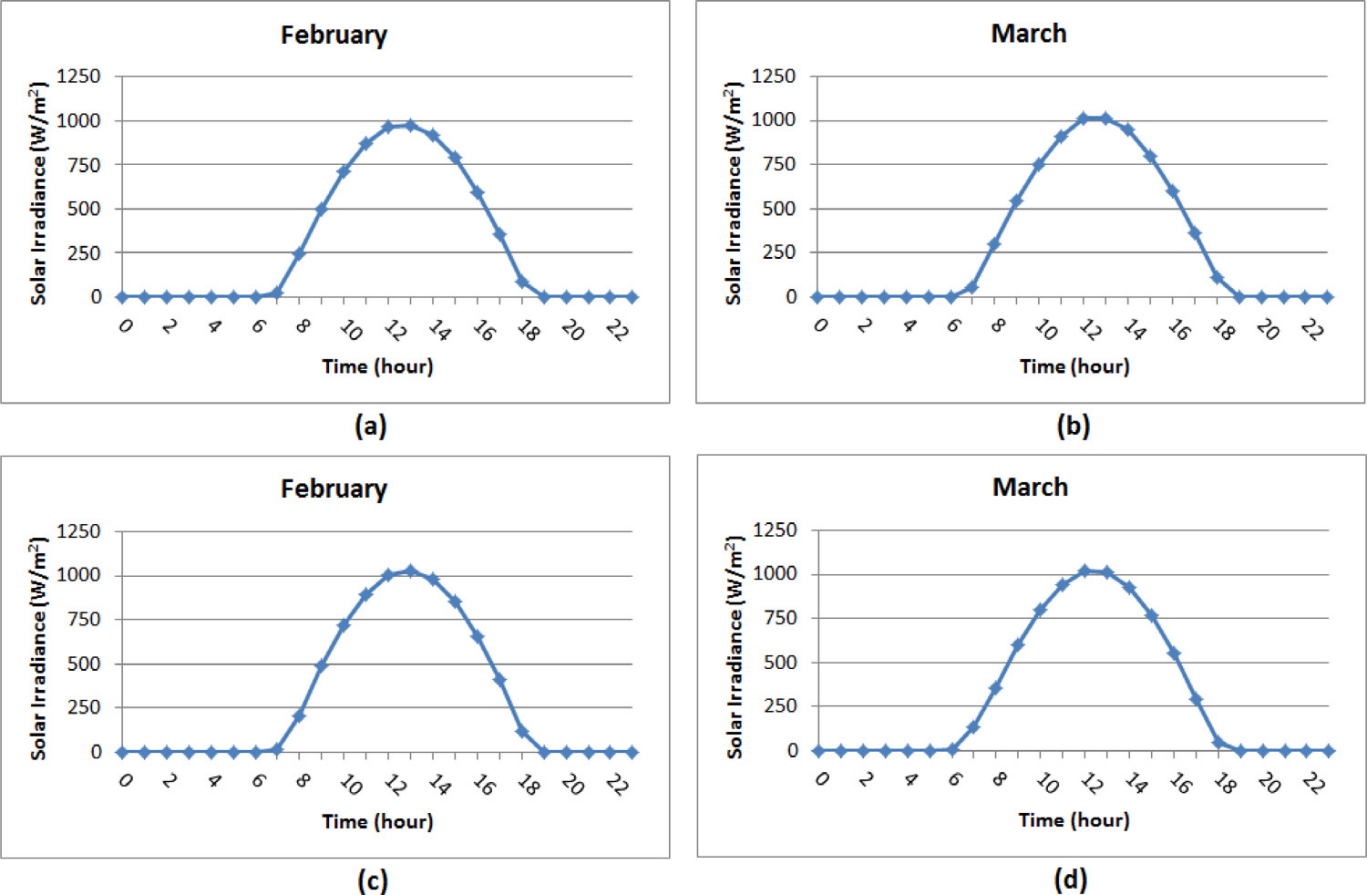
Average hourly solar radiation (W/m^2^) for typical days of dry months in 2012: (a) Dera, (b) HS, (c) MD, (d) MU campus.

**Fig. 9:**
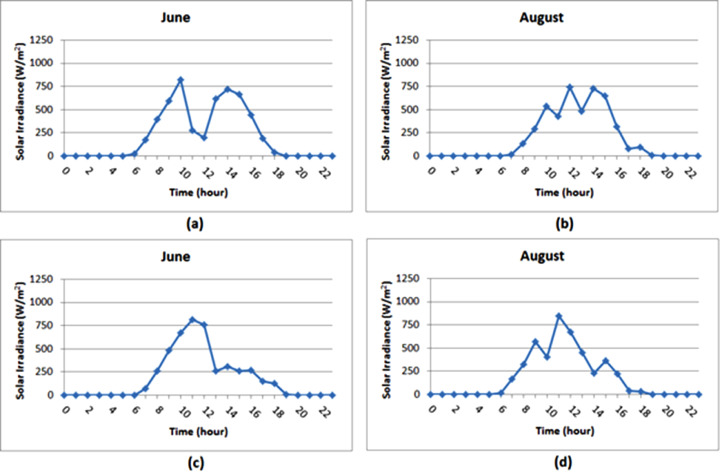
Average hourly solar irradiance (W/m^2^) for typical days of rainy months in 2012: (a) Dera, (b) HS, (c) MD, (d) MU campus.

**Fig. 10:**
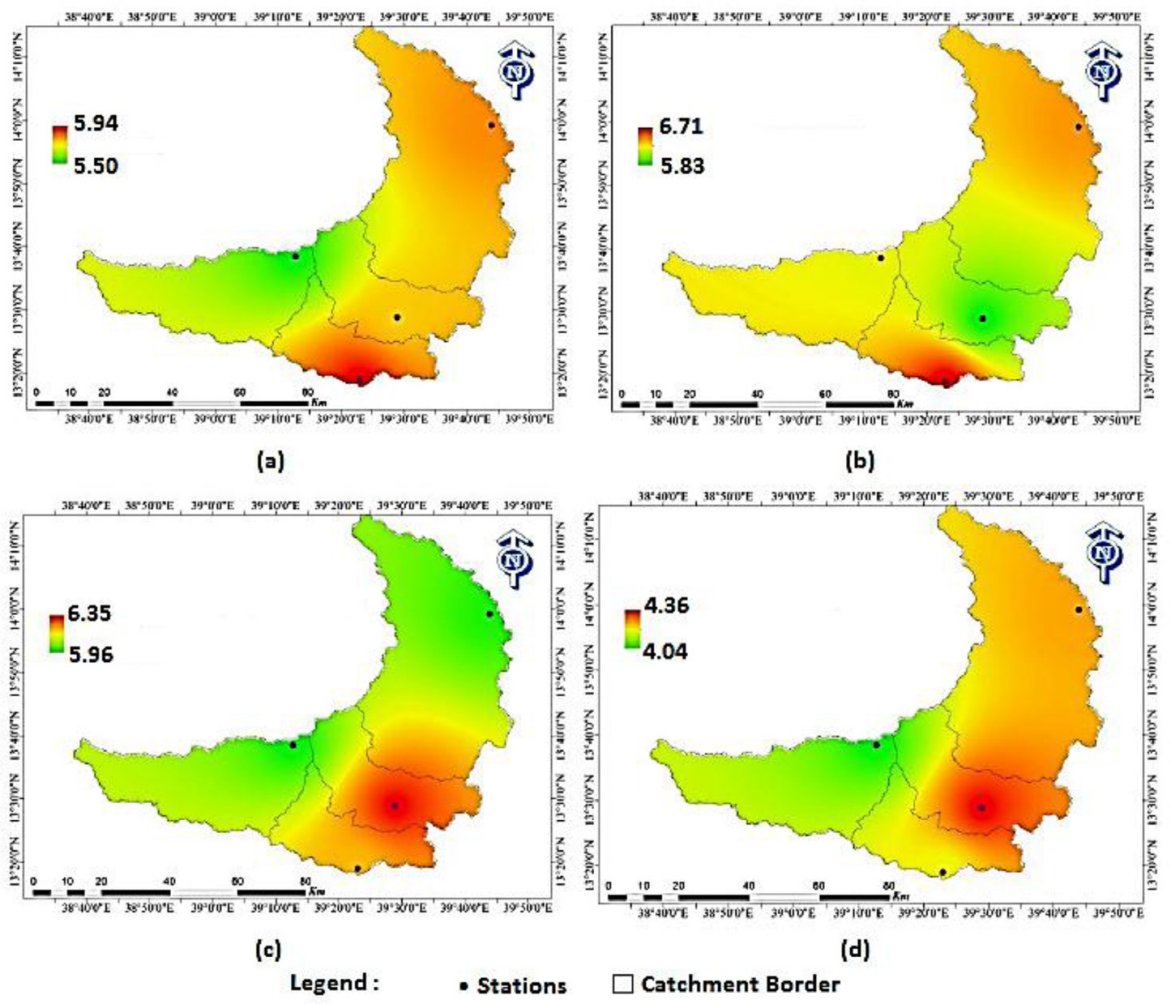
Seasonal average daily solar irradiation (kWh/m^2^) distribution (a) autumn, (b) winter, (c) spring, (d) summer [2].

The measurement instruments at the four sites include:

• Ekopower model EKO21 N Data Logger Code 178, 179, 275, 276, respectively. Code 178 failed and was replaced by a new Code 314 on January 19, 2012.
• Max40+ Anemometer
• DIR21+ Wind Vane
• Davis Vantage Pro 6450 Solar Radiation Sensor

The logging set up was the default set up: three seconds interval, 200 data points, and ten-minute average data were recorded in the data logger. Instruments were checked after installation for accurate readings. To check for the proper operation of the instruments, a visit to the site was made a few days after installation. Once confirmed that the system was functioning properly, the instruments were set up to log continuously. Regular visits were made to the site to check the instrumentation.

### 2.3 Data collection and processing

The recording was carried out at ten minutes intervals continuously. Data were downloaded to a laptop regularly to avoid any loss of data in case of faults in the instrumentation. The ten-minute time interval data were inspected for completeness and missing data were identified. The raw data collected from the data logger was processed in three steps: (i) Data extraction, (ii) Data Qualification and Labeling, and (iii) Data Analysis.

Initially, the raw data that existed as a text file was exported to a Microsoft Excel spreadsheet to simplify the task of the data analysis. The raw data was extracted from the text data into Excel as a “delimited” with a semicolon (;) and colon (:). The semicolon is column delimiter and the colon is to have the hour, minute, and seconds in separate columns for further ease of analysis. The extracted data contains measurements logged every 10 min and the Excel sheet is saved with a file name RawSitename_MonthYear_MonthYear.xls.

After exporting the raw data, careful data qualification and labeling were done. The RawSitename_MonthYear_MonthYear.xls file was open and saved with a different name to keep the raw data for any further check-up (filename: QLSitename_MonthYear_MonthYear.xls). QL stands for qualified and labelled data file. To have the correct labels on the Excel sheet, headers (Date, Hour, Minute, Second, and Solar radiation) were inserted at the first row of the worksheet. In addition, an assessment to remove errors or uncertainty that may lead to biased and misleading results was done. All unnecessary and outlier data which are out of the objective were discarded according to the study conducted by Zell et al. in 2015 [4]. The time series number of the count was checked to identify missing, negative, invalid, and outlier data points. Range (Min-Max) validation was carried out to ensure the largest and the lowest value within the pyranometers limits (0–1800 W/m^2^). Since complete time series data is important for performance prediction to ensure the reliability of the system, missing data were replaced using an averaging of six preceding data. During the long period of measurement gap, the period is noted and no averaging of the data was carried out. However, the large degree of the gap in data was canceled without filling its missed value. Then, a validated data file has been created in Microsoft excel. The collected data per ten minutes interval was expected to have consisted of 6 data per hour or 144 data per day. The total annual data expected from the sites were calculated by multiplying by the number of hours or number of days in a year. To combine the QLSitename_MonthYear_MonthYear.xls Excel sheets into one, the data points are copied and pasted into a new file saved with a file name QLdata_Geba catchment.xls*.* The file includes radiation values with headers (Date, Time, and Site name_Year).

To perform the temporal and spatial variation of solar radiation in the Geba catchment, a time series analysis was conducted using the ten-minute aggregated data from each of the four sites. Based on the ten-minute interval raw data, hourly average, diurnal average, monthly average daily, three months average or seasonal average daily, and annual average daily radiation were statistically determined. The calculation procedure was done using Excel Ku tools advanced sort and functions in Excel. Open the QLdata_Geba catchment.xls file and save it with a new name. File name DAdata_Geba catchment.xls. DA stands for data analysis.

The hourly average solar irradiance (W/m^2^) was calculated by employing the AVERAGE function on the Excel worksheet based on the six data points in a given hour. The hourly data was saved in a new worksheet and named HDA_Geba catchment.xls for further analysis. The hourly data represents the basis for calculating the diurnal averages. Similarly, the SUBTOTAL function was employed to calculate the daily solar irradiation (Wh/m^2^) based on the 24 h data points of a given day. The solar irradiation was much more useful in the process of data reduction and simplifying the difficulty of rapid changes along with the weather condition. From the daily solar irradiation data, the average and range (minimum and maximum values) for the respective months and years were analyzed. The daily irradiation represents the basis for calculating the monthly-averaged daily, seasonally-averaged daily, and annually-averaged daily radiations.

The spatial distribution and potential of solar radiation in the catchment were analyzed using geographical coordinates (longitude and latitude) of each measurement station and average solar radiation that has been prepared by ArcGIS 10.1. The distribution of the solar radiation in the catchment was interpolated using the ArcGIS spatial interpolation technique, inverse distance weighting (IDW) interpolation method with a spatial resolution of 30 m × 30 m [5]. Following interpolation, the ArcGIS spatial analyst tool was used to reclassify the average solar radiation values. The Symbology tool was also used to plot color maps to show the spatial variation in the catchment [4]. The technical solar energy potential was obtained based on the analysis of five criteria: solar radiation availability, land cover, slope, distance to the national road, and distance to the transmission line. The technical solar energy potential was calculated by excluding areas not suitable for solar farms within the defined boundaries [6]. Finally, the findings were cross-checked against other results obtained from other studies to develop conclusions and recommendations.

## CRediT authorship contribution statement

**Mulu Bayray:** Conceptualization, Methodology, Data curation, Supervision, Writing – review & editing. **Yacob Gebreyohannes:** Conceptualization, Methodology, Supervision, Software, Writing – original draft, Writing – review & editing. **Hailay Gebrehiwot:** Investigation, Software, Validation. **Solomon Teklemichael:** Supervision. **Anwar Mustefa:** Conceptualization, Methodology, Data curation. **Asfafaw Haileslassie:** Conceptualization, Methodology, Data curation. **Petros Gebray:** Conceptualization, Methodology, Data curation. **Ashenafi Kebedom:** Conceptualization, Methodology, Data curation. **Fana Filli:** Conceptualization, Methodology, Data curation.

## Declaration of Competing Interest

The authors declare that they have no known competing interests.

## Appendix Supplementary materials

Image, application 1

Image, application 2

## References

[1] Mahmud A.M. (2014). Solar energy resource assessment of the geba catchment, Northern Ethiopia. Energy Procedia.

[2] Bayray M. (2021). Temporal and spatial solar resource variation by analysis of measured irradiance in Geba catchment, North Ethiopia. Sustain. Energy Technol. Assess..

[3] Zenebe A. (2013). Spatial and temporal variability of river flows in the degraded semi-arid tropical mountains of Northern Ethiopia. Z. Geomorphol..

[4] Zell E. (2015). Assessment of solar radiation resources in Saudi Arabia. Sol. Energy.

[5] Aguilar F.J., Agüera F., Aguilar M.A., Carvajal F. (2005). Effects of terrain morphology, sampling density, and interpolation methods on grid DEM accuracy. Photogramm. Eng. Remote Sens..

[6] A. Lopez, B. Roberts, D. Heimiller, N. Blair, and G. Porro, US Renewable Energy Technical Potentials. A GIS-Based Analysis. National Renewable Energy Lab.(NREL), No. NREL/TP-6A20-51946 (2012). 10.2172/1219777.

